# Diagnosis of Infantile Diseases

**Published:** 1886-04-20

**Authors:** Benjamin A. Bradley

**Affiliations:** Cincinnati, Ohio


### DIAGNOSIS OF INFANTILE DISEASES.

BY BENJAMIN A. BRADLEY, M. D., CINCINNATI, OHIO.

Congestion of the cheeks of an infant (except in cachetic and prostrated states of the system) denotes febrile or inflammatory diseases.

Transient circumscribed congestion of the face, ears, forehead, strabismus (with febrile reaction), oscillation of iris, irregularity of the pupil, dropping of the upper lids, denotes cerebral disease.

Rapid wasting of the features, causing suborbital depressions with prominence and pointedness of the cheek bones, denotes diarrhœal affections.

Great emaciation occurring gradually, denotes subacute or chronic troubles of grave character.

Downward direction of the axis of the eyes, with smallness of the face and great expansion of the cranium, denotes chronic hydrocephalus.

Enlargement of the spongy portion of the bones denotes rhachitis.

Bulbous enlargement of the fingers and curving of the finger nails denotes cyanosis.

A thick meibomian secretion of a puriform appearance between the eye-lids denotes a state of great depression.

Passive congestion of the vessels of the conjunctiva denotes the near approach of death.

Protracted lividity, and lividity produced by exertion or excitement while the respiration is normal, denotes malformatiom of the heart or vessels.

Temporary lividity denotes severe acute diseases.

Absence of tears in infants, four or even over four months old, denotes a fatal form of disease.

Sharp piercing cry denotes severe cerebro-spinal diseases.

Irregular muscular movements, partially controlled by the will during consciousness, denotes choera.

Contraction of the eyebrows, turning eyes and head from light, denotes headache.

Carrying the hand to the ear, laying the head against the breast of the mother or nurse, denotes earache.

Carrying the fingers to the mouth with fretfulness, denotes some disease of the larynx.

Rubbing or pressing the nose, denotes worms or some other intestinal disease.

Moving from side to side denotes some obstructive disease of the larynx.

Hoarse or indistinct voice denotes laryngitis.

Feeble or plaintive voice denotes .disease of the abdominal organs.

Perfect quietude, sunken features, much changed by smilling or crying, denotes exhausting diarrhœal affections.

Respirations slow, intermittent and accompanied by sighing, denotes cerebral disease.

Respirations slight and accelerated denotes inflammatory diseases of the larynx and trachea.

Respirations intermittent and accelerated, more than in other diseases, denotes capillary bronchitis.

Cough loud and sonorous denotes spasmodic croup.

Cough hoarse and harsh denotes true croup.

Cough clear and distinct denotes bronchitis.

Cough surpressed and painful denotes pneumonia and pleurisy.

Cough convulsive denotes whooping cough.

Cough dry and painless, as sometimes found in typhoid and intermittent fevers, difficut dentition, and worms; occuring in such diseases, it is sometimes dependent on more or less bronchitis to which the primary disease has given rise.—*Medical Call.*

